# Community perspectives on access to maternal health services during the COVID-19 pandemic in rural Western Kenya: a qualitative study

**DOI:** 10.1186/s12913-025-13162-1

**Published:** 2025-08-23

**Authors:** Neema Kaseje, George Agango Odhiambo, Beldine Omondi, Andy Haines, Meghna Ranganathan

**Affiliations:** 1 Surgical Systems Research Group, PO Box 4074, Kisumu, Kenya; 2 London School of Hygiene & Tropical Medicine, Keppel Street, London, WC1E 7HT UK; 3 Tropical Institute of Community Health, PO Box 4074, Kisumu, Kenya

**Keywords:** COVID-19 pandemic, Mothers, Maternal health, Community health workers, Rural Kenya

## Abstract

**Background:**

Globally, maternal and child health outcomes were negatively affected during the COVID-19 pandemic. There is limited qualitative evidence focused on access to maternal health services during the COVID-19 pandemic in rural sub-Saharan African populations. This study aims to fill this gap by exploring key community perspectives on access to maternal health services during the COVID-19 pandemic in rural western Kenya.

**Methods:**

We conducted five focus group discussions and sixty one in-depth interviews. Participants were mothers who delivered in 2020 during the acute phase of the COVID-19 pandemic and rural community health workers (CHWs) in Siaya and Kisii counties. The three-delay model framework helped to guide discussions around access to maternal health services during the COVID-19 pandemic in rural western Kenya. We transcribed the data and conducted a thematic content analysis.

**Results:**

According to CHWs and expectant mothers, fear and misconceptions about COVID-19 were associated with delays in making the decision to seek maternal health care. Lockdowns, movement restrictions, and curfews made it challenging to physically reach health facilities. The shortage of drugs and supplies and an insufficient number of healthcare workers in health facilities were barriers to care seeking. The quality of maternal health services was perceived to have declined during the COVID-19 pandemic. Mothers reported the *Linda Mama* health insurance program helped them pay for maternal health services during the COVID-19 pandemic; however, out-of-pocket expenses were common. As reported by mothers, CHWs when engaged and active, helped alleviate access challenges by serving as a link to the health system. There were significant socio-economic difficulties experienced by community members because of closed businesses and schools.

**Conclusions:**

Community perspectives revealed significant challenges with accessing maternal health services during the COVID-19 pandemic in rural western Kenya. The pandemic amplified each of three delays in accessing care leading to poorer access to maternal health services. The overriding perception among mothers was that CHWs helped mitigate challenges around access to health services. CHWs should be included in future pandemic preparedness and response efforts.

**Supplementary Information:**

The online version contains supplementary material available at 10.1186/s12913-025-13162-1.

## Background

According to World Health organization (WHO) estimates, the COVID-19 pandemic led to 775,830,200 infections and 7,056,108 deaths worldwide [1]. Moreover, the COVID-19 pandemic had broader consequences for the use of health services: a systematic review of 81 studies across 20 countries demonstrated a 42% reduction in visits to health facilities; a 28% reduction in hospital admissions; a 31% reduction in diagnostics tests; and a 30% reduction in therapeutic interventions [2]. Globally, the COVID-19 pandemic had a negative impact on maternal and child health (MCH) services utilisation and subsequent MCH outcomes [3, 4]. A study across 18 countries demonstrated a 2.6% reduction in the use of maternal health services [3]. In parts of Asia, such as in India, there were reductions in institutional deliveries and a reduction in the provision of emergency obstetric care, particularly during the first lockdown period [5]. In Malaysia, there were reductions in the use of antenatal care, contraception, and postnatal care [6].

We observed poorer utilisation of MCH services in sub–Saharan Africa (SSA) during the COVID-19 pandemic. In Burundi, a comparative analysis of skilled birth attendance (SBA) before the COVID-19 pandemic and during the initial phase of the COVID-19 pandemic demonstrated a 85% reduction in SBA between April 2020 and April 2019 [7]. In south-west Ethiopia, there were reductions in the use of antenatal care services, facility-based deliveries, and immunisation services by 27.4%, 23.5%, and 28.5% respectively [8]. Furthermore, in a multi-country study of eight countries in SSA, decreases in postnatal care services ranged from 10 to 25% [9].

The reduction in the use of maternal health services during the COVID-19 pandemic was linked to poorer MCH outcomes. In Brazil, Carvalho-Sauer et al. observed a 33.4% increase in the maternal mortality rate (MMR) and a 3.2% increase in the perinatal mortality rate [4]. According to WHO, MMR is defined as the death of a woman during pregnancy, childbirth, or within 42 days of pregnancy termination from any cause related to the pregnancy or its management [10]. MMR is reported as the number of maternal deaths per 100,000 live births [10]. A second study reported a 38.6% increase in the maternal mortality rate and a 44.7% increase in the child mortality rate across 118 low and middle-income countries as a result of increased healthcare needs and delayed access to care [11].

Following global patterns, reduced MCH services utilisation in SSA was linked to poorer MCH outcomes during the COVID-19 pandemic. The average MMR increased from 342 per 100,000 to 542 per 100,000 [12, 13] in sharp contrast to the progress that was being made in SSA before the COVID-19 pandemic. Before the COVID-19 pandemic, countries in SSA were on track to significantly reduce MMR; from 2000 to 2017, MMR in SSA had reduced by 38% [12]. Key factors that contributed to worsening maternal health services utilisation and maternal health outcomes in SSA during the COVID-19 pandemic include reduced demand for maternal health services and delayed access to maternal health services due to health systems that were overwhelmed with COVID-19 cases [13, 14].

Existing qualitative evidence suggests poorer access to maternal health services and negative maternal experiences during the COVID-19 pandemic. An evidence synthesis by Flaherty et al. including 48 qualitative studies involving women and maternity care providers, demonstrated that maternity care during the COVID-19 pandemic was negatively experienced by both women and maternity care providers [15].

Rural health systems in SSA were particularly vulnerable to MCH service disruptions and poorer MCH outcomes during the COVID-19 pandemic because of weaker health systems with fewer health care workers and fewer health facilities compared to urban counterparts at baseline before the pandemic was declared [16, 17]. Moreover, poor connectivity and communications infrastructure in rural SSA make telehealth solutions for maternal health services difficult to implement [18]. Thus, we chose to focus on rural communities because they are more vulnerable to disruptions and lack of continuity of care at baseline; and are consequently more prone to major health system disruptions during pandemics.

A systematic review of the literature shows multiple gaps in the current evidence. First, the current evidence on disruptions in MCH services utilisation, and deterioration in MCH outcomes during the COVID-19 pandemic focuses predominantly on quantitative data [19]. Second, there is limited qualitative evidence addressing rural populations and mothers who delivered in rural SSA during the COVID-19 pandemic. Only two out of the 48 studies included in Flaherty’s et al.’s qualitative evidence synthesis were from SSA (Kenya and Ethiopia); and neither study included mothers from rural western Kenya [15].

### The three delays model

The Three Delays model was initially proposed by Thaddeus and Maine (1994) to identify indirect factors that contributed to maternal deaths [20]. The model identifies three critical phases that have direct impact on the survival of the baby and the mother namely: delay in the decision to seek care (first delay); delay in identifying and reaching the health facility (second delay); and delay in receiving care at the health facility (third delay) [20]. The three-delays model is often cited as an important framework for describing and analysing access to maternal health services; however, the model has faced some criticism namely: it has not been comprehensive enough in addressing continuity of care; it does not capture comorbidities such as HIV/AIDs which are important contributors to maternal deaths [21, 22]. Furthermore, the model does not take into consideration primary prevention or early disease detection; and it does not emphasise the role of the community and its collective ability to mobilise resources. In addition, the model may focus too much on care seeking and not enough on the quality of services when community members reach health facilities [21, 22]. Lastly, the social status of women is a concern that can influence agency, and their ability to make decisions for themselves [21, 22]. In response to these limitations, revisions of the model have been proposed. For instance, Bhutta et al. revised the model by breaking down the first delay into 2 components (delay in recognition, and delay in decision making) - and they found that 34% of women experienced delays in decision making [22]. We drew on aspects of the three-delays model to further understand the use of maternal health services during the COVID-19 pandemic in rural western Kenya. We sought to explore how the different delays would manifest themselves during the COVID-19 pandemic.

Given the evidence gaps mentioned earlier, the main objective of this study was to explore key community perspectives around accessing MCH services during the COVID-19 pandemic in rural western Kenya.

## Methods

### Study setting

The study was conducted in two counties in rural western Kenya -- Siaya and Kisii. Siaya is a rural county in Western Kenya with a population of 993,183. Siaya has 240 community health units (CHUs); with approximately 10 CHWs covering each CHU [23]. Kisii is also a rural county in Western Kenya with a population of 1,266,860. It has 291 CHUs, with approximately 10 CHWs covering each unit [23].

The CHW intervention was implemented in Siaya only. In Kisii county, the standard Kenya national COVID-19 protocol was implemented and did not include a CHW intervention.

### Community health worker intervention

Under the leadership of Siaya Ministry of Health (MOH), the intervention focused on training, equipping, and deploying CHWs to reduce COVID-19 infections and deaths in Siaya. CHW training included raising awareness about COVID-19, and preventing and detecting COVID-19 cases. Furthermore, CHWs in Siaya were trained on how to isolate and manage minor and moderate cases of COVID-19 at home [23]. To ensure optimal management of COVID-19 cases at home, CHWs were trained on how to digitally monitor and report vital signs at the household level [23]. In addition, Siaya CHWs were trained to ensure households continued to use essential health services including maternal and child health services during the COVID-19 pandemic [23]. Following the training, CHWs in Siaya were equipped with KN95 masks, thermometers, and pulse oximeters; and they subsequently visited households in Siaya [23]. During household visits, Siaya CHWs educated households about COVID-19 prevention; they screened and isolated potential COVID-19 cases in households; and they referred severe cases of COVID-19 to health facilities with oxygen capacity [23].

Kisii and Siaya counties in western Kenya were selected because at baseline, both counties have both poorer socioeconomic indicators and poorer maternal health outcomes compared to national indicators [24, 25]. As a result, Kisii and Siaya counties would be more vulnerable to maternal health services disruptions and potentially poorer maternal health outcomes during a pandemic. In addition, at baseline, the health system in western Kenya is severely under-resourced in terms of staffing, supplies, and equipment; which contribute significantly to the third delay in accessing care. This study was part of a larger study, and the CHW intervention was implemented only in Siaya [23].

### Study design

To capture lived experiences of women who delivered during the COVID-19 pandemic and CHWs who supported households in rural western Kenya, we used the phenomenological qualitative design adopting the descriptive approach. Phenomenological qualitative design seeks to describe the essence of a phenomenon by exploring it from the perspective of those who have experienced it [26]. Given the novelty of COVID-19, this was an important approach to adopt as we sought to explore perceptions, interpretations, and understanding of women seeking maternal health services during the COVID-19 pandemic and CHWs who supported households during the COVID-19 pandemic. During data collection and analysis, we aimed to set aside our own preconceptions and biases to better understand participants’ perspectives. To minimise bias, data collection and analysis were conducted by team members who were not involved in the intervention in Siaya. In addition, to limit selection bias and researcher bias, we used CHWs to identify women participants who delivered babies in 2020 because of their high population coverage rates. Reported CHW population coverage rates for health interventions in western Kenya are as high as 91% versus 40% of the population that access hospital care [27, 28]. Using hospital-based registries would have excluded those who did not deliver in hospitals and likely experienced more barriers to access.

### Sampling strategy and participants

We used purposive sampling methods as described by Kuzel and Morse, and the three-delay model framework to guide discussions around access to maternal health services during the COVID-19 pandemic in rural western Kenya in addition to access barriers identified in the literature [20, 29, 30]. We used the three delays model to inform our interview guides and questions asked during the IDIs and FGDs. In addition, we used the three delays model to construct the conceptual framework which guided our thematic analysis.

Because our study design adopted the phenomenological approach which focuses on exploring and highlighting lived experiences; our sampling method used CHWs closely connected with households in western Kenya to identify women who gave birth during the COVID-19 pandemic in 2020. This approach was more likely to generate a sample of women representative of the community compared to sampling that depended solely on the research team or the hospital based team who may be considered outsiders and may only have contact with those of higher socioeconomic status for instance. Using CHWs to identify women who delivered during the pandemic allowed us to minimise researcher selection bias, and the selection bias inherent in hospital based registries which would exclude women who did not deliver in health facilities.

We conducted focus group discussions (FGDs) and in-depth interviews (IDI) in the intervention county (Siaya) and the comparison county (Kisii county) to explore perspectives on access to maternal health services during the COVID-19 pandemic in rural western Kenya.

Participants interviewed included mothers (aged 17 to 43 years old) who delivered in 2020 during the acute phase of the COVID-19 pandemic, as well as CHWs. In Kisii county, we conducted two FGDs and 33 IDIs with CHWs (*n* = 10) and mothers (*n* = 23) who delivered in 2020. The first FGD was composed of five male CHWs, five female CHWs, and two community health extension workers (CHEWs). The second FGD was composed of eight female CHWs, two male CHWs, and 2 CHEWs.

In Siaya county, we conducted three FGDs and 28 IDIs with CHWs (*n* = 8) and mothers (*n* = 20) who delivered in 2020. The first FGD was composed of five female CHWs and one male CHW. The second FGD in Siaya comprised seven mothers and one CHW (all respondents were female). The third FGD was composed of seven female CHWs and three male CHWs.

### Ethical considerations

We obtained ethical approval from the London School of Hygiene and Tropical Medicine (LSHTM, reference number 27252) and the Jaramogi Oginga Odinga Teaching and Referral Hospital (JOOTRH, approval number IERC/JOOTR/219/20). Before each interview, we obtained written informed consent from each participant. Each participant received information about the study and were allowed to ask questions, and their questions were responded to Consent was obtained in English, Swahili, or Luo depending on the participant and what language they were most comfortable speaking.

### Data collection procedure

The recruitment of participants was done in multiple steps. First, we met with Siaya and Kisii county Ministries of Health to discuss objectives and planned activities. Second, we coordinated with CHWs to identify women who delivered in 2020 during the COVID-19 pandemic. Third, CHWs communicated with mothers who delivered in 2020 and asked them if they would be willing to participate in discussions regarding their perspectives and experiences delivering in 2020 during the COVID-19 pandemic. CHWs were instructed to reach out to all mothers who had delivered babies in 2020 in their respective catchment areas. Fourth, CHWs assisted with bringing mothers to interview sites for FGDs and IDIs.

We conducted FGDs and IDIs in pairs: one person conducted the interview while the second person took notes. Interviews were recorded using a digital voice recorder. Recordings were stored in a secure icloud repository. The person conducting the interview ensured that all participants had an opportunity to speak and prompted those that had not spoken to share their views during FGDs. Interviews lasted 40 to 60 min. Data were collected from August 2022 to October 2022. The interview guide we used is included as a Supplementary file 1.

All data collectors received appropriate training from experienced researchers in the techniques of conducting FGDs and IDIs including principles of qualitative research, the objectives of the current study, FGD facilitation techniques, interviewing techniques, and ethics and informed consent. In addition, they received training in the interview guide and the three delays model framework. Data collection was conducted in CHW meeting locations which were a convenient meeting point for mothers and CHWs. In addition, we conducted interviews in homes ensuring confidentiality was maintained during interviews. All data collectors were proficient in English and Kiswahili, and two out of three were proficient in Luo.

### Reflexivity/positionality statement

The lead author (NK) was involved in the implementation of the CHW intervention in Siaya and comes from western Kenya. Consequently, it was important to minimise potential biases that could have influenced findings in this study. On the one hand, because NK had worked with CHWs and communities in Siaya, it was easier to develop a rapport with CHWs and mothers during data collection. This may have facilitated the discussion of difficult topics during FGDs and IDIs. On the other hand, a potential disadvantage of being involved in the Siaya CHW intervention was that perspectives regarding the intervention may have been presented in an overly positive light, and raising concerns about the intervention may have been difficult for respondents. To mitigate these potential biases, data collection and analysis were conducted with team members who were not involved in the Siaya intervention and were not from Siaya or Kisii counties. Moreover, an additional factor that may have influenced our findings is the fact that the lead author is a trained surgeon, and it was not typical for the lead author to be interacting with mothers, CHWs, and other stakeholders in communities. As a result, both the lead author and respondents may have experienced nervousness as they interacted with each other. Furthermore, both parties likely had to make adjustments in how activities proceeded. For instance, flexibility in the timing of interviews was necessary in order to capture as many respondents as possible. This is in contrast to a more regimented schedule that is typical of operating room theaters that the lead author was used to. Having diverse members of the research team helped with navigating adjustments that were needed to fully explore community perspectives and experiences. In addition, having the lead author, a member of the hospital team, in the community likely helped with creating a safe space for mothers and CHWs to voice their perspectives and concerns in their own environment. They likely do not experience the same liberty to voice their opinions when in health facilities.

### Approach to analysis

FGDs were used to get preliminary themes on perspectives and experiences of mothers and CHWs. As previously described, FGDs use group dynamics to stimulate responses among group members and allow the emergence of insights by participants sharing and questioning views that are discussed [31]. Emerging themes were further explored during in-depth interviews (IDIs) using the three-delay model as a preliminary framework for guiding discussions. As previously described, IDIs allow for further exploration of participant experiences, their perceptions, beliefs, and attitudes, regarding maternal health services during the COVID-19 pandemic in rural western Kenya.

Following data collection and transcription, line-by- line coding was conducted as a first step to allow for close examination of the data. This step allowed the researchers to gain an initial understanding of participants’ experiences and perspectives. We defined codes as described by Charmaz [32]. Key emerging themes were extracted and an inductive deductive thematic analysis was conducted as previously described by Miles and Huberman using Nvivo [33, 34]. The inductive deductive approach allowed for initial codes to be developed; following subsequent reviews of the data, additional codes were added as they emerged. We identified emerging themes and subthemes and presented them in narrative form according to respondents’ roles and the location of the respondents. Following this step, we summarised and synthesised the data. In summary, we analysed our data in five distinct steps: (i) close examination of the data by reviewing transcripts line by line; (ii) development of a coding framework; (iii) identification of themes and subthemes; (iv) organisation of themes and subthemes in narrative form according to respondents’ roles and locations; and (v) synthesis and final interpretation of the data.

The overall goal in our thematic content analysis was to identify recurring themes expressed by CHWs and mothers who delivered during the COVID-19 pandemic in rural western Kenya. Following the transcription of interviews, we coded and grouped related concepts to identify overarching themes. We conducted an iterative process of familiarising ourselves with the data, developing initial codes, grouping codes into themes and interpreting the results. To familiarise ourselves with the data we read and reviewed transcripts to gain an understanding of the content. Next, we conducted the initial coding and identified concepts which were assigned to transcripts. To develop themes, we grouped related codes together ensuring they captured recurring issues in the data. In the final step, we interpreted identified themes by explaining the meaning of identified themes and providing supporting evidence.

## Results

At baseline Kisii county had a higher maternal mortality rate (MMR) compared to Siaya county; both MMRs were higher than the average national MMR of 130 per 100 ‘000 live births justifying our focus on maternal health services and maternal health outcomes in rural western Kenya [24]. In addition, Table 1 shows that Siaya and Kisii counties had similar baseline standardised mortality per 100 ‘000 population and life expectancy rates in years. The literacy rate was higher in Kisii county compared to Siaya county; however both rates were lower than the reported national literacy rate of 78.8%% [25]. Despite Kisii women potentially having a higher socioeconomic status than women in Siaya as indicated by higher rates of literacy and house ownership; delivery in health facilities and delivery by a skilled provider were similar in both counties at baseline. Table 1 below shows health and sociodemographic indicators in Siaya and Kisii counties.

**Table 1:** Description of health and sociodemographic indicators in Siaya and Kisii counties [24, 35]

Health and sociodemographic indicator	Siaya county	Kisii county
Life expectancy (years)	64.7	67
Baseline standardised mortality/100’000	1190	1140
Maternal mortality rate/100’000 population	202.4	347.7
Literacy rate %	33.7	48.3
Delivery in health facilities %	69.6	69.3
Delivered by a skilled provider %	70.4	72.8
Women with no house ownership %	48	43.3

The results below are organised by key themes that emerged during our analyses, namely: (i) the fear of COVID-19; (ii) the lack of material and human resources and perceived poor quality of maternal health services; (iii) the role of CHWs during the COVID-19 pandemic; (iv) socioeconomic hardship during the COVID-19 pandemic; and (v) transport challenges due to lockdowns, curfews, movement restrictions, high cost of travel, and bad roads. Key themes are further organised to align with the three delays model framework.

### First delay

Fear of COVID-19 contributed significantly to the first delay - the delay in making the decision to seek care.

#### Fear of COVID-19

Kisii mothers and CHWs described in great detail the fear of COVID-19 that prevailed among community members and health workers, during the COVID-19 pandemic. There was fear of the disease itself and how it affected the physical condition of community members, which led to a fear of contracting COVID-19 and the potential death and disability that could come with an infection. Because of the fear of COVID-19 community members avoided going to health facilities for health services including routine health services.

*“People feared going to the hospital for fear of contracting COVID-19 or being hospitalised for symptoms related to COVID-19.” (CHW 9 Kisii county)*.

*“Most people were unwilling to go to the hospital.” (CHW 6 Kisii county)*.

Fear of COVID-19 was ubiquitous across the community in Kisii. The fear was present even among healthcare workers who were on the frontlines dealing with patients presenting with COVID-19, non COVID-19 conditions, and emergencies.

*“There was a time even health care providers were not at work because of fear of COVID-19.” (CHW 4 Kisii county)*.

Similarly, mothers in Kisii also expressed their fear of COVID-19 during the pandemic. There was worry about the severity of the disease, which prompted expectant mothers to deprioritise the need to attend antenatal care clinics.

*“I was afraid to attend clinics due to fear of being infected.*” *(Kisii county mother 10)*.

When expectant mothers weighed the risks linked to missing an antenatal care visit, and the risk of contracting COVID-19, they felt that it was safer to miss routine antenatal care visits than to attend a clinic and potentially contract COVID-19 that could be life threatening. Missing an antenatal care visit was not immediately life threatening.

Expectant mothers who contracted COVID-19 observed fear among healthcare workers who were in the hospital treating them. They felt they were not treated well because of the fear experienced by healthcare workers. In particular, healthcare workers avoided close contact with patients who had COVID-19 because they themselves were afraid of getting COVID-19.

*“We were not treated well because everyone feared they may be infected*,* even the nurses would not associate themselves with us.” (Kisii county mother 15 diagnosed with COVID-19)*.

Siaya CHWs made similar observations to Kisii CHWs; they also observed the fear of COVID-19 among expectant mothers who subsequently avoided attending antenatal clinics in health facilities to reduce their risk of getting COVID-19. The fear was rooted in the fact that COVID-19 was a new disease.

“*It was a new disease. Women avoided facilities for fear of getting infected.” (Siaya county CHW 1)*.

Siaya CHWs also observed that service delivery across multiple disease entities was affected by the pandemic. Not only were routine health services such as the use of antenatal care services affected, but long-standing programs such as tuberculosis programs experienced reductions in the use of services during initial phases of the COVID-19 pandemic.

*“Initially patients going for TB services*,* facility deliveries*,* and first ANC visits went down. Later community members learned that they could go to health facilities wearing masks and the fear of health facilities is now gone.” (Siaya county CHW 7)*.

### Second delay

Transport challenges due to lockdowns, curfews, movement restrictions, high cost of travel, and bad roads contributed significantly to the second delay during the COVID-19 pandemic in rural western Kenya.

Expectant mothers experienced significant challenges with transport during the COVID-19 pandemic. These challenges were particularly acute in Siaya - where mothers walked to facilities to deliver their babies and walked back home after delivery. Curfews and movement restrictions led to fewer public transportation options by taxi or motorbike (boda boda). One expectant mother who was unable to travel to the hospital because of the lack of transport during curfew hours delivered at home.

*“It was much harder to get pregnant women to hospitals. There was one home delivery who did not make it to the hospital on time because of the lack of transport; there was a curfew at that time.” (Siaya county CHW 5)*.

Most public transportation operators were not willing to travel during curfew hours because they risked being stopped by policemen who were patrolling roads to ensure movement restriction rules were being followed by community members.

*“There was curfew in place and no one was willing to travel at night.” (Siaya county mother 11)*.

Some transport operators were willing to help mothers despite the curfew rules.

*“We found a Probox driver who was willing to assist us but at a higher cost because he was risking breaking curfew rules.” (Siaya county mother 18)*.

An intervention that helped justify transportation during curfew hours was the presentation of a letter written by health facilities which documented that an expectant mother may go into labor and that they should be allowed safe passage to reach the hospital.

*“Facilities writing a letter for patient movement made a difference.” (Siaya county CHW 7)*.

High costs of transportation to hospitals made taking taxis and boda bodas too expensive, and the only option left for expectant mothers in Siaya was to walk to the hospital.

*“When I went into labor I walked to the hospital because I did not have enough money for transport.” (Siaya county mother 14)*.

*“I had to walk for a distance to reach the hospital because there were no motorcycles or a vehicle to offer transport services.” (Siaya county mother 5)*.*“A boda boda was 100 to hospital and 100 back home which was expensive.” (Siaya county mother 9)*.

In addition to curfews, increases in transportation prices, and fewer public transportation options, roads particularly in Siaya were difficult to navigate during rains, and expectant mothers were forced to wait until it stopped raining to start their journey to the health facility.

*“It was raining and the roads were not in good state*,* and we had to wait.” (Siaya county mother 4)*.

### Third delay

Being an under-resourced health system with an insufficient number of healthcare workers, insufficient supplies, and consumables, contributed to the third delay and to poorer quality of maternal health services during the COVID-19 pandemic in western Kenya. In addition, during the pandemic, the third delay was magnified due to additional shortages in staffing, supplies, and consumables linked to the significant lack of PPE, COVID-19 therapeutics, and COVID-19 vaccines in SSA. SSA was the last region to receive COVID-19 vaccines which were critical to protecting the lives of health care workers. Moreover, health system resources such as space/hospital beds and antibiotics were redirected to the COVID-19 response. There was a national directive requiring each county to have a minimum number of COVID-19 beds.

During the COVID-19 pandemic, there were serious concerns among all respondents that there was a lack of supplies, drugs (including antibiotics), and healthcare workers.

*“There were no drugs in the hospital*,* you could be attended to by a nurse and be told to go and buy drugs at the chemist.” (Kisii county mother 21)*.

These gaps were noted in both public hospitals and private hospitals; however, public hospitals seemed to experience more stockouts in supplies and drugs.

*“There were no drugs in public hospitals so most of the people went to private hospitals.” (Kisii county mother 13)*.

As a result of stockouts in supplies and drugs, expectant mothers were forced to, either choose a private facility that was likely to be better equipped, or proceed with a public facility and buy the necessary drugs and supplies needed to deliver. Table 1 shows sociodemographic indicators of women in Siaya and Kisii counties.

“*When mothers get to the health facility*,* they have to buy everything*,* they have to buy the medicine*,* if they need any antibiotics or any other medication.” (Siaya county CHW 1)*.

*“I spent money from my pocket to buy cotton wool*,* soap and a basin.” (Siaya county mother 7)*.

There was also a lack of space particularly in public facilities; expectant mothers were forced to share beds; some were turned away from facilities and had to seek care elsewhere because of the lack of space.

“*I went to the hospital but was turned away since there was no space left to accommodate another pregnant mother - mothers were sharing beds.” (Siaya county mother 18)*.

The lack of space, drugs and supplies was not the only challenge. Once mothers reached health facilities, there was an inadequate number of healthcare workers which resulted in healthcare workers being overwhelmed.

*“During my time of delivery*,* there were few health care providers.” (Kisii county mother 6)*.

Healthcare workers were not only taking care of expectant mothers but also of emergencies, COVID-19 cases, and other health conditions requiring hospitalisation.

*“Only one doctor was on the shift and was serving both the maternity wing and managing other cases.” (Siaya county mother 7)*.

In the most extreme form, healthcare workers were absent during the healthcare worker strike in Siaya which was driven by the lack of personal protective equipment (PPE) for healthcare workers. As a result of the strike, no maternal health services were available.

*“I reached the hospital at 1:00AM*,* once I reached the hospital I was turned away because doctors were on strike and no services were available at the hospital.” (Siaya county mother 16)*.

In addition, because of the strike, expectant mothers were forced to visit multiple facilities before they were able to access maternal health services.

*“I was delivered at Rabuor private hospital because there was a strike at Yala hospital.” (Siaya county mother 17)*.

Furthermore, because of the lack of healthcare workers there was a reliance on nursing and clinical officer students to provide maternal health services.

*“When it was time for delivery there were only KMTC (Kenya Medical Training College) students around and they were the ones who helped me deliver.” (Siaya county mother 6)*.

Expectant mothers reported experiencing complications from receiving care during the COVID-19 pandemic - specifically, a postoperative wound infection that was associated with cotton being left in the wound during a Cesarean section.

*“After a period of four weeks*,* I realised I was not healing and actually pus had started flowing out of my wound. When I went to the hospital it was devastating - cotton had remained in my wound.” (Kisii county mother 8)*.

Similarly, CHWs reported observing a decline in the quality of services available in health facilities during the COVID-19 pandemic.

*“There were more challenges with getting quality health services*,* and also more and more out of pocket expenses. (Siaya county CHW 5)*

### The role of CHWs during the COVID-19 pandemic in mitigating access barriers and the three delays during the COVID-19 pandemic and improving health outcomes 

Actions taken by trained and equipped CHWs mitigated the three delays to accessing maternal health services during the COVID-19 pandemic in rural western Kenya.

How communities experienced the presence of CHWs during the COVID-19 pandemic was not uniform across counties. In one county, the presence of CHWs was very limited during the COVID-19 pandemic. CHWs in Kisii county reported they did not feel they could visit homesteads with expectant mothers. They reported that community members were afraid of them and afraid of contracting COVID-19. Furthermore, CHWs in Kisii county reported feeling ill equipped to conduct household visits during the COVID-19 pandemic because they did not have the proper protective equipment. In addition, they reported feeling they did not have the necessary training in COVID-19 - a new disease. They also reported feeling a lack of recognition of their services during the COVID-19 pandemic.

*“We did not have the protective gear so it was hard to attend to sick people*,* and we were not allowed into people’s houses because everyone feared contracting the disease.” (Kisii county CHW 3)*.

*“CHWs should be empowered through acknowledgement of the services they render; we should be trained regularly and given the required support.” (Kisii county CHW 8)*.

In parallel, expectant mothers in Kisii county experienced the relative lack of presence of CHWs in Kisii county; they report that CHWs were not visiting them during the COVID-19 pandemic. Expectant mothers described the need to have more self agency and to make their own health decisions given the relative absence of CHWs in Kisii county.

*“During that time the CHW was not visiting the homestead as required*,* so it was a personal initiative to take care of myself.” (Kisii county mother 13)*.

In contrast to CHWs in Kisii county, CHWs in Siaya county were highly engaged during the COVID-19 pandemic. The Siaya Ministry of Health (MOH) sought them to conduct surveillance activities within their community health units to identify, report, isolate, and monitor potential cases of COVID-19 during the pandemic.

*“We participated in surveillance*,* notifying the MOH of possible cases.” (Siaya county CHW 7)*.

*“We participated in contact tracing. We identified potential cases*,* followed them*,* and isolated them. We are the unsung heroes.” (Siaya county CHW 6)*.

Siaya CHWs were tasked with educating households in the community about COVID-19; how it was transmitted and how it should be managed. Siaya CHWs came across misinformation, including within churches, and they took action to convey the correct information so that community members and community leaders were well informed about COVID-19.

*“We were able to get the right information*,* and give households the right information about COVID-19 symptoms*,* prevention*,* and care.” (Siaya county CHW 3)*.

*“There were instances where churches passed on the wrong information*,* and dialogues were held*,* so that everyone had the right information.” (Siaya county CHW 4)*.

Not only were CHWs involved in COVID-19 related activities, but they also continued promoting access to maternal health services during the COVID-19 pandemic.

*“We mapped all pregnant women; each had an individual delivery plan*,* which includes their EDD (estimated delivery date)*,* who will accompany them*,* the facility where they will deliver*,* and a transport plan (numbers of boda bodas (motorbike operators) and 100–200 shillings saved).” (Siaya county CHW 2)*.

Siaya CHWs felt confident enough to function as doctors in the community with ready access to households. Siaya CHWs felt confident managing COVID-19 cases in households when the isolation and management of COVID-19 cases were shifted away from health facilities to households.

*“We acted as doctors in the community and community members including those on home based care were accountable to us.” (Siaya county CHW 8)*.

When community members recovered from COVID-19, CHWs were available to reintegrate them into society by addressing the stigma that was associated with COVID-19 infections. CHWs were there to reassure community members that it was safe to be around someone who had recovered from COVID-19.

*“We helped address stigma and helped patients re-enter society after recovery from COVID-19. Communities have a lot of trust in us.” (Siaya county CHW 3)*.

When COVID-19 vaccines became available; CHWs were present to educate community members about the benefits of COVID-19 vaccines; they also functioned as role models for community members by being the first ones to be vaccinated to show community members that COVID-19 vaccines were safe.

*“We were able to convince community members to get vaccinated by talking to them*,* again*,* and again.” (Siaya county CHW 7)*.

In contrast to expectant mothers in Kisii county, expectant mothers’ in Siaya county felt that CHWs were available to them during the COVID-19 pandemic, and were present in their homes where they conducted household visits. During these household visits, CHWs in Siaya reminded expectant mothers to attend their antenatal clinic appointments. They ensured mothers were accountable and checked with them that they had attended their clinics. CHWs in Siaya were also available during emergencies. In a case where a mother was in labor and was walking to the hospital because they did not have means of transport, the CHW was available to assist the mother and the newborn to reach the hospital safely.

*“I gave birth while walking to the hospital. We immediately called the CHW*,* and they helped us reach the hospital.” (Siaya county mother 8)*.

### Economic hardship during the COVID-19 pandemic contributed to the three delays in accessing maternal health services 

Community members, expectant mothers, and CHWs experienced significant economic challenges during the COVID-19 pandemic.

In addition, community members including expectant mothers had difficulty paying for health services which contributed to the first, second, and third delays in accessing maternal health services during the COVID-19 pandemic.

*“I lacked funds to manage myself at the hospital*,* I was required to have supplies like cotton wool.” (Siaya county mother 12)*.

Some expectant mothers had to borrow funds to pay for Cesarean sections.

*“I gave birth through CS and was told to pay in cash which I didn’t have - I had to borrow from a friend.”(Siaya county mother 4)*.

Other mothers used Linda mama to pay for maternal health services; however, there was variability on whether Linda mama was accepted by health facilities during the COVID-19 pandemic.

*“I used Linda mama for payment of the bill” (Kisii county mother 19)*.

*“We were told that Linda Mama was not accepted*,* and we were to pay a total amount of Ksh 30*,*000 to be released” (Siaya county mother 16)*.

From a broader social standpoint, schools were closed and school closures had a significant impact on young girls who were vulnerable to teenage pregnancies.

Adolescent girls were affected by school closures; many fell pregnant during the COVID-19 pandemic.

*“I was a student at Kisumu Polytechnic when I got pregnant. I had to stop my studies*,* and I have not continued with my studies since” (Siaya county young mother 10)*.

## Discussion

In this study, we explored perspectives and experiences of mothers who delivered babies in 2020, and CHWs to further understand the availability and use of maternal health services during the COVID-19 pandemic in rural western Kenya. Community perspectives and experiences revealed significant challenges with access to maternal health services during the COVID-19 pandemic in rural western Kenya. Mothers and CHWs reported delayed access to care and poorer quality of care driven by the fear of COVID-19, misconceptions about COVID-19 and COVID-19 vaccines, transport challenges, and shortages of drugs, supplies, and healthcare workers. Furthermore, there were significant socioeconomic challenges due to closed businesses and schools which were associated with more poverty and increased teenage pregnancies. Mothers reported using the government sponsored *Linda Mama* insurance program to pay for maternal health services during the COVID-19 pandemic in rural western Kenya with variable success. When actively engaged, CHWs mitigated maternal health services access challenges in rural western Kenya.

### Fear of COVID-19 as a barrier to accessing maternal health services

The fear of COVID-19 was likely present because COVID-19 was a new disease with many unknowns. Early on, the scientific community was still learning about COVID-19, its virulence, its transmission patterns, and its management [36]. Because of the many unknowns, community members and health care workers did not want to expose themselves to COVID-19 particularly in hospitals. The lack of PPE exacerbated the situation for healthcare workers who felt they were not adequately protected and were in real danger of contracting COVID-19 and potentially dying from COVID-19 [37].

Fear of COVID-19 likely led to reduced access to maternal health services in rural western Kenya during the COVID-19 pandemic by potentiating the three delays described by Thaddeus and Maine [20]. In addition, because of fear of COVID-19 among healthcare workers, contact with patients was kept to a minimum, likely leading to poorer quality of maternal health services provided. In a qualitative study in south eastern Kenya, Ombere et al. also found that fear of COVID-19 among mothers in Kilifi county was a key barrier to accessing maternal health services and was associated with an increase in the incidence of home births in Kilifi county during the COVID-19 pandemic [38]. Fear of COVID-19 with reduced access to maternal health services during the COVID-19 pandemic was also reported by Graham et al. [39].

### Transport challenges due to lockdowns, curfews, movement restrictions, high cost of travel, and bad roads

In addition to reduced demand for maternal health services due to fear of COVID-19, health facilities were physically less accessible because of lockdowns, movement restrictions, curfews, and fewer means of transport options during the COVID-19 pandemic. This barrier was particularly evident in Siaya, where women frequently walked to reach health facilities and one even gave birth while walking to the hospital. Similar findings were noted in West African countries including Nigeria and Ghana - where women had difficulty reaching health facilities because of curfews that were implemented to reduce the transmission of COVID-19 [12, 40]. In addition, Bick et al. and Mekonnen et al. reported similar access challenges due to movement restrictions in Uganda, Tanzania, Kenya, and Ethiopia [18, 41]. Transport challenges experienced by pregnant women in rural SSA predated the COVID-19 pandemic and could have been predicted to continue or even worsen during the COVID-19 pandemic. Transport challenges were experienced more acutely in Siaya where the county surface area is greater and distances traveled are greater [42–44]. In future pandemic preparedness and response efforts, provisions should be made to mitigate transport challenges experienced by expectant mothers making attempts to use maternal health services in rural SSA.

### Lack of material and human resources and poor quality of maternal health services

There were likely shortages of drugs, supplies, and healthcare workers because health resources were shifted towards the COVID-19 response to the detriment of baseline population health needs [13, 14]. Furthermore, perspectives from mothers indicate that the quality of maternal health services was poor during the COVID-19 pandemic. There are several reasons for why the quality of maternal health services may have deteriorated during the COVID-19 pandemic. First, the health system was overburdened because it was dealing with both COVID-19 cases and routine health services including maternal health services. Second, inadequate supplies and drugs are key drivers of poor quality of care [45]. Third, overworked healthcare workers and poor communication by healthcare workers likely contributed to poor quality of maternal health services. According to Afulani et al. four factors influence women’s perceptions of quality of care: responsiveness, supportive care, dignified care, and effective communication [45]. When seeking maternal health services, women have positive experiences when they are received well at the health facility, treated with kindness and respect, and given sufficient information about their care [45]. The reverse leads to a negative experience [45]. The current evidence shows that key drivers of poor quality of care include inadequate training, the lack of drugs, supplies, equipment, and healthcare workers, and poorly motivated healthcare workers [46]. In our study, the lack of drugs, supplies, and healthcare workers was reported by both mothers and CHWs in both counties and likely drove poor quality of maternal health services in rural western Kenya during the COVID-19 pandemic.

Additionally, poor communication among health care workers often leads to poorer quality of care. For example, cotton wool was left in a young mother’s wound; optimised healthcare worker communication may have reduced the chances of this happening during intra-operative instrument and sponge counts as recommended by the WHO surgical safety checklist [47]. Discrepancies in numbers and the communication of discrepancies would have prompted the team to search further for the missing cotton wool. Similar to our findings, Semaan et al., found changes in the quality of maternal health services in SSA during the COVID-19 pandemic; the quality of care deteriorated during lockdowns and travel bans [47, 48]. In addition, quality control measures such as maternal death reviews were postponed or stopped which likely contributed to poorer quality of maternal health services during the COVID-19 pandemic [47, 48].

### Socioeconomic hardship during the COVID-19 pandemic

The economic hardship experienced by community members was likely a result of lost jobs and businesses which increased poverty during the COVID-19 pandemic [49]. A World Bank study across 49 countries, found that women-led businesses were more severely affected by the COVID-19 pandemic than those led by men [50]. Compounding the problem of poverty was the rise in prices for basic needs including food and transport. Economic hardship had an impact on access to maternal health services because transportation to health facilities was too expensive for mothers. Mothers also had to buy drugs and supplies because these were not available in hospitals. Oyugi et al. also report out of pocket expenses in Kiambu county (central Kenya): mothers paid an average of 9.5$ for a normal delivery and 10.88$ for a Cesarean Sect. [51]. A second study including five counties in Kenya reported that mothers continued to incur out-of-pocket expenses despite being enrolled in the Linda Mama program [52].

There was increased social isolation during the COVID-19 pandemic which likely had a negative impact on the mental health of community members including an expectant mother who was isolated after being diagnosed with COVID-19. Isolating COVID-19 patients may have contributed to more anxiety and/or depression during the COVID-19 pandemic. Oluoch-Aridi et al. found that insufficient social support was a significant risk factor for depression among pregnant women in Kenya [53]. Increased isolation and poverty among community members may explain CHW observations of increased violence during the COVID-19 pandemic. Furthermore, in Siaya, especially, there was displeasure with how funerals were conducted which community members felt went against social norms of gathering many friends and family members to mourn the loss of loved ones. Community members felt COVID-19 burial directives were a second trauma in addition to losing a loved one. In future pandemic preparedness and response efforts, it would be important to get community input as directives are formulated to limit the disconnect between public health measures that are necessary and cultural norms and practices.

Contributing factors to the rise in teenage pregnancies during the COVID-19 pandemic in Kenya included school closures and poverty [54]. These factors have been highlighted by a WHO report which cites termination of education, reduced job and career prospects, and increased vulnerability to poverty and exclusion as key factors that may lead to increased teenage pregnancies [55]. Alunyo et al. report similar findings in Uganda; where they observed a 13.9% increase in the teenage pregnancy rate following the first COVID-19 lock down in 2020 [56].

### The role of CHWs during the COVID-19 pandemic

CHWs were active during the COVID-19 pandemic when adequately trained and supported because adequate training, equipping, and support, led to increased CHW knowledge, skill, and credibility. These findings align with our previously published quantitative data showing that training and equipping CHWs was associated with increased CHW household visits, fewer COVID-19 infections, and fewer COVID-19 deaths as result of improved community member linkages with the health system [23]. In a second study from Pakistan, CHWs also improved linkages between pregnant women and the health system for abortion and contraception services during the COVID-19 pandemic [57]. This intervention allowed women to maintain access to these services during the COVID-19 pandemic [57].

### Application of the three-delay model during a pandemic

The three-delay model was developed during non-pandemic times [20]. Therefore, there are important considerations to be added to the framework to optimise its applicability in rural SSA during a pandemic. First, in addition to reduced demand for maternal health services due to fear of COVID-19, the potential to convert demand into use of services was severely diminished because of economic hardship and transport challenges due to lockdowns, curfews, and poor road networks. Consequently, delay two was a major barrier to care in rural sub-Saharan Africa during the COVID-19 pandemic. Moreover, because of healthcare worker strikes, and an overburdened health system that had insufficient supplies, drugs, and a health workforce, delay three was magnified in this context. Pandemic preparedness and response efforts need to adapt to these differences and implemented solutions should address highly pronounced delays one, two, and three to mitigate poor access to MCH services and poor MCH outcomes during pandemics in rural SSA. Thus, we propose that the three-delays model should be modified to be a dynamic framework with different degrees of delays to reflect baseline delays in access (during non-pandemic times) and amplified delays in access to maternal health services (during pandemics and disease outbreaks) [20].

**Fig. 1:**
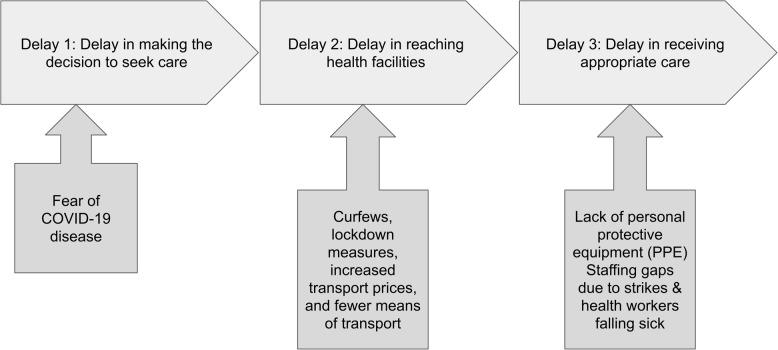
The proposed modified three delays model highlights emerging themes not included in the original three delays model, specifically: fear of COVID-19; movement restrictions, increases in transport prices; the lack of PPE and staffing shortages

The critical finding from this study was that mothers experienced greater barriers and greater delays when accessing maternal health services during the COVID-19 pandemic. Important themes emerged that were specific to the COVID-19 pandemic and historically have not been included in the three delays model, such as: curfews, movement restrictions, and the need for PPE to improve the safety of accessing health services during a pandemic that was highly infectious with documented human to human transmission. Thus, we propose a modified three delays model which takes into account important pandemic specific factors to improve its applicability in rural western Kenya during the COVID-19 pandemic (Fig. 1).

### Strengths and limitations of our study

Our study has several strengths. First, our study offers insights that are relevant to rural communities in sub–Saharan Africa. 70% of Kenya is rural - and therefore rural perspectives are important when planning future pandemic preparedness and response efforts in Kenya and other sub-Saharan countries [58]. Second, our study has perspectives from young mothers in rural western Kenya who are often not included in health discussions and health interventions. Third, we propose modifications to the three-delay model to increase its applicability in rural SSA during pandemics. Fourth, our study highlights key areas that will require interventions in future pandemic preparedness and response efforts.

Our study had several limitations: first, we used purposive sampling that may have increased the possibility of selection bias among our respondents. Second, our findings may not be applicable in urban settings and outside of sub-Saharan Africa. Third, there could have been some recall bias among respondents; interviews took place a year and a half after the COVID-19 pandemic was declared. In addition, the majority of respondents were women, hence the male perspective was underrepresented in our results. Future studies may seek to obtain perspectives from male respondents to explore how their active participation can potentially support pregnant women who need maternal health services during a pandemic. An additional limitation was that we did not determine the socioeconomic status (SES) of individual study participants because this may have reduced participation. SES is a significant factor in determining maternal health outcomes [59]. More studies are needed to further explore the role of SES in accessing MCH services during pandemics in rural western Kenya. In the past, wealth indices using asset and livestock ownership and access to electricity and clean water have been used to determine socio-economic status in rural Kenya; these variables were beyond the scope of our current study [60]. Lastly, the first author was involved in implementing the CHW intervention in Siaya. To ensure objectivity, data collection and analysis were also conducted by team members who were not involved in the intervention in Siaya. Furthermore, key themes that emerged were corroborated by evidence available in the literature providing external validity to key findings in this study.

### Policy and programming implications

Policy measures should focus on mitigating the three delays recognising that during a pandemic the three-delay model may have more unrealised demand for services in addition to supply challenges at the health system level. CHW interventions should be implemented to maintain demand for maternal health services and to promote the conversion of the demand for maternal health services into the use of maternal health services during a pandemic. In rural contexts, transportation should be included in pandemic preparedness and response efforts to reduce significant delays in reaching health facilities. At the health system level, it is critical to ensure adequate supplies, drugs, and a workforce. In the Kenyan context, the Linda Mama program needs to be supplemented during pandemics because it did not cover all expenses linked to accessing maternal health services; out-of-pocket expenses increased during the COVID-19 pandemic at a time when poverty also increased. Quality of services suffered during the pandemic and there should be specific interventions, to train, and supplement healthcare workers when routine health services need to be provided in addition to services related to the pandemic. A cohort of trained CHWs can serve this purpose, particularly with home-based care interventions. It is important to monitor and continue quality control measures to ensure that the quality of maternal health services do not suffer during pandemics. In the setting of a new disease, it is critical to rapidly educate community members and healthcare workers to minimise fear and the emergence of misinformation and disinformation which can significantly reduce access to care. Policy makers should prioritise education and raising awareness with the right information from trusted sources.

In addition, more interventions are needed to protect young mothers during pandemics including the ability to rejoin education following pregnancies. In the future, there should be standardised policies that ensure young mothers are able to return to school following delivery with adequate support from family members and schools.

In conclusion, community perspectives and experiences revealed significant challenges with accessing maternal health services during the COVID-19 pandemic in rural western Kenya. The three delays were magnified leading to reductions in the use of maternal health services. The government sponsored Linda Mama insurance program helped mothers pay for maternal health services during the COVID-19 pandemic in rural western Kenya; however, out of pocket expenses remained significant. Lastly, when actively engaged, trained and equipped CHWs mitigate maternal health access challenges and CHWs should be formally included in pandemic preparedness and response efforts.

## Supplementary Information

Supplementary Material 1.

## Data Availability

No datasets were generated or analysed during the current study.

## Abbreviations

CHEWs: Community Health Extension Workers
CHWs: Community Health Workers
COVID-19: Coronavirus disease 2019
EDD: Estimated Delivery Date
IDIs: In-depth interviews
KMTC: Kenya Medical Training College
FGDs: Focus group discussions
MCH: Maternal and child health
MMR: Maternal mortality rate
MOH: Ministry of Health
PPE: Personal Protective Equipment
SBA: Skilled birth attendance
SSA: Sub Saharan Africa
WHO: World Health Organization
