# Supplementary material for: Community perspectives on access to maternal health services during the COVID-19 pandemic in rural Western Kenya: a qualitative study
Source: BMC Health Serv Res. 2025 Aug 23;25:1122. doi: 10.1186/s12913-025-13162-1 (PMC12374302; doi:10.1186/s12913-025-13162-1)
Supplement: Supplementary file 1 — Supplementary Material 1. [file 12913_2025_13162_MOESM1_ESM.docx]

***Semi-structured interviews and focus group discussions guiding questions:***

Community health workers:

Describe your experience providing health services during the pandemic?

How did things change during the pandemic?

Describe any challenges or successes you encountered?.

Community members (mothers):

Describe your experience getting health services during the pandemic?

In addition, we used the Three delays model to further explore community perspectives (19). The Three delays model identifies three critical phases that have direct impact on the survival of the baby and the mother namely: delay in the decision to seek care (first delay); delay in identifying and reaching the health facility (second delay); and delay in receiving care at the health facility (third delay) (19).
